# Midterm Outcomes of Four-Corner Fusion Surgery Using Nitinol Staples

**DOI:** 10.1016/j.jhsg.2025.100805

**Published:** 2025-08-07

**Authors:** Arya A. Ahmady, Mohammad Zalzaleh, Barth B. Riedel

**Affiliations:** ∗ Department of Orthopaedic Surgery, Loma Linda University, Loma Linda, CA

**Keywords:** Four-corner fusion, SLAC, SNAC, Wrist arthritis, Wrist fusion

## Abstract

**Purpose:**

Wrist arthritis because of scaphoid nonunion advanced collapseand scapholunate advanced collapse can be treated through scaphoid excision and four-corner arthrodesis. There are many fixation techniques; however, there are few studies reporting outcomes in which only Nitinol staples were used. This study aimed to evaluate whether patients undergoing a four-corner arthrodesis using this fixation technique as well as intraoperative modifications to minimize complications will have successful outcomes.

**Methods:**

Retrospective study of patients who underwent scaphoid excision and four-corner arthrodesis using DynaNite Nitinol Staple (Arthrex Inc) to treat scaphoid nonunion advanced collapse or scapholunate advanced collapse wrist arthritis at a single academic institution by a single surgeon. The technique and our modifications are described below. Outcome measures included radiographic union, range of motion, strength, and patient-reported outcomes such as Quick Disabilities of the Arm, Shoulder, and Hand, Michigan Hand Outcomes questionnaire, Likert patient satisfaction scale, and the visual analog scale.

**Results:**

Eight patients were included with mean follow-up of 27 months (9–42 months). When compared to the contralateral side, patients maintained 76% wrist flexion-extension, 83% radial-ulnar deviation, and 99% pronation-supination. Key pinch, three-point pinch, and grip strength testing were 95%, 89%, and 86%, respectively. The mean Quick Disabilities of the Arm, Shoulder, and Hand score was 17, and the mean Michigan Hand Outcomes score was 85% compared to the contralateral side. The visual analog pain scale had a mean of 2.5 (range 0–6). Radiographic imaging showed that all patients had undergone union of their four-corner arthrodesis with intact hardware.

**Conclusions:**

The results of this study show that midterm outcomes of four-corner fusions performed with Nitinol staples have promising results in overall functional and patient-reported outcomes. In addition, they offer reliable fixation for achieving union of the four-corner arthrodesis.

**Type of study/level of evidence:**

Therapeutic IV.

Although the wrist joint does not experience the weight bearing force of the ankle or knee, it still experiences considerable weight bearing while performing everyday activities. Constant use of this joint predisposes it to trauma, degeneration, and arthritis.^1^ A systematic review of 4,000 wrist radiographs showed osteoarthritis in 5% of wrists, with involvement near the scaphoid in over 95% of cases, thus demonstrating the high prevalence of wrist arthritis.^2^ There are various surgical treatments for this condition, however the specific type of surgery depends on the etiology and the location of the arthritis. Surgical treatment options include posterior interosseous nerve denervation, radial styloidectomy, complete or partial carpal excision, proximal row carpectomy, capitolunate arthrodesis, four-corner arthrodesis, and complete wrist arthrodesis.

Scapholunate advanced collapse (SLAC) and scaphoid nonunion advanced collapse (SNAC) are the two most common patterns of posttraumatic wrist arthritis.^3^ With chronic scapholunate ligament injury and scaphoid nonunions, progressive osteoarthritis can occur in a predictable fashion, ultimately leading to pancarpal arthritis.^4^ Management of the later stages of wrist arthritis secondary to SLAC and SNAC can be achieved with scaphoid excision and four-corner arthrodesis, particularly when the arthritic changes involve the radioscaphoid and lunocapitate articulations and spare the radiolunate articulation. Although studies have demonstrated that fusion rates of 90% can be achieved independent of fixation technique, there are several important complications to consider regarding selecting a technique for an individual patient.^5^ K-wire fixation can be associated with pin track infections (which occur in 6% of cases) as well as pin migration irritating soft tissues. Locking plates carry the risk of dorsal impingement and screw breakage, while headless compression screws demonstrating increased risk of progression toward radiolunate arthritis, even 80% in some cases.^6^ Some studies demonstrated patients treated with headless compression screws have improved arc of motion and lower complication rates than those treated with staple fixation, whereas others suggest that these difference may not clinically relevant all the while the union rates remain similar.^7^^,^^8^ Of these methods, there are few studies reporting midterm outcomes of patients in which Nitinol staples were used as the sole fixation method.

The DynaNite Nitinol Staple (Arthrex Inc) staples are unique implants that exhibit memory properties, allowing them to apply constant compression between their legs over time when heated to physiological temperatures.^9^ In addition, the modulus of elasticity of the staples has been shown to be closest to that of bone than any other metal used in the body.^10^ In theory, these properties will allow bone to heal faster without creating a stress riser. Therefore, this implant should be a reliable option when fusion of bony ends is desired. However, there have been concerns regarding potential complications of staples such as dorsal impingement from the implant as well as concern for a bicortical leg length of the staple being within the carpal tunnel.^11^ A biomechanical study proposes modifications to minimize some of these complications. One of these modifications includes creating a trough in the lunate–capitate interface for the staple prior to insertion to minimize dorsal implant prominence, thus preventing impingement with extension. They demonstrate that this modification, as well as not placing bicortical staples, does not impact the biomechanical properties of nitinol staples; however, these modifications have yet to be seen on a larger clinical scale.^9^ This study seeks to determine whether patients undergoing a four-corner arthrodesis for wrist arthritis secondary to SLAC and SNAC using DynaNite Nitinol Staple (Arthrex Inc) as well as the proposed technical modifications will have successful clinical and radiographical outcomes.

## Materials and Methods

This was a retrospective case series performed at a single academic institution from 2016-2021 (IRB Approval Number: IIRR-01300).

Patients included in the study were those who underwent scaphoid excision and four-corner arthrodesis surgery using DynaNite Nitinol Staple (Arthrex Inc) for the management of SLAC or SNAC wrist arthritis. Patients who were unable to understand or participate in the study, those who previously had surgery on either wrist, or those with a baseline dysfunction of either upper extremity were excluded from the study. Overall 21 patients were initially selected and eight patients met the inclusion criteria, completed the questionnaires, and had appropriate follow-up.

After approval by the institutional review board, Current Procedural Terminology codes (25210, 25825) were used to search the electronic medical records for patients who qualify for the study. Patients who met the inclusion criteria were contacted via telephone and invited to the office to participate in the study. Objective measures such as wrist range of motion, grip strength, and pinch strength were measured by the surgeon and or resident physician. Patients also obtained X-rays of the operative wrist, and then completed the Quick Disabilities of the Arm, Shoulder, and Hand (*Quick*DASH) questionnaire, Michigan Hand Outcomes questionnaire, Likert Satisfaction Scale to gauge overall satisfaction with surgery, and a visual analog pain scale to assess the current pain level.^11^^,^^12^ Patients were compensated for their time with a $100 generic gift card.

**Figure 1:**
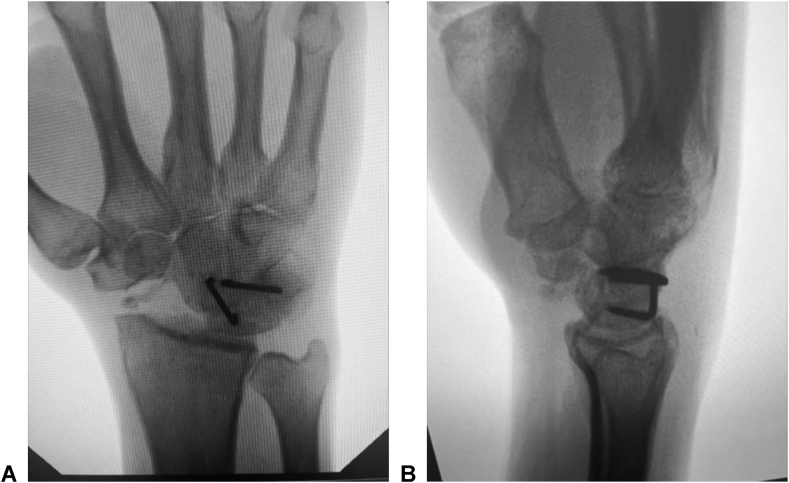
**A** Posterior-anterior X-ray of wrist after completion of four-corner fusion using this construct. **B** Lateral X-ray of wrist after completion of four-corner fusion using this construct.

The data were collected and organized into a spreadsheet, including basic patient demographics, diagnosis and months since surgery. The degree of motion for both wrists were measured with a goniometer, which included wrist flexion and extension, radial and ulnar deviation, pronation, and supination. The mean arc of motion was then calculated followed by the percent difference between the operative and nonsurgical extremity (Table 1). Strength in pounds was measured bilaterally for key pinch, three-point pinch, and grip strength with a dynamometer. The mean strength was calculated as well as the percent difference between the operative and nonsurgical extremity. Mean scores were calculated from the *Quick*DASH questionnaire, Michigan Hand Outcomes questionnaire, Likert Satisfaction Scale, and the Visual Analog Scale. The data were analyzed for considerable differences between range of motion, strength, and patient-reported outcomes based on the Michigan Hand Outcomes questionnaire. Radiographs were then assessed for successful fusion and confirmed with the radiology report for any signs of arthritis or nonunion of the fusion mass in addition to any problems with the hardware (Fig. 1).

**Table 1:** Demonstration of Range of Motion of Each Participant in the Study After Surgery

Participant	Flexion (Operative)	Flexion (Nonsurgical)	Extension (Operative)	Extension (Nonsurgical)	Flexion-Extension Arc (Operative)	Flexion-Extension Arc (Nonsurgical)	Flexion-Extension Arc Difference
1	30	60	30	50	60	110	50 (54.5%)
2	40	50	15	15	55	65	10 (84.6%)
3	50	60	40	70	90	130	40 (69.2%)
4	40	40	25	45	65	85	20 (76.5%)
5	30	70	30	35	60	105	45 (57.1%)
6	45	50	65	70	110	120	10 (91.7%)
7	45	55	40	35	85	90	5 (94.4%)
8	50	50	45	65	95	115	20 (82.6%)
Mean	41.25	54.375	36.25	48.125	77.5	102.5	25 (76.3%)
*P* value							0.011

Participant	Radial Deviation (Operative)	Radial Deviation (Nonsurgical)	Ulnar Deviation (Operative)	Ulnar Deviation (Nonsurgical)	Radial-Ulnar Deviation Arc (Operative)	Radial-Ulnar Deviation Arc (Nonsurgical)	Radial-Ulnar Deviation Arc Difference

1	15	20	20	45	35	65	30 (53.8%)
2	5	10	20	20	25	30	5 (83.3%)
3	20	30	50	50	70	80	10 (87.5%)
4	15	20	45	45	60	65	5 (92.3%)
5	10	20	45	50	55	70	15 (78.6%)
6	20	20	30	40	50	60	10 (83.3%)
7	20	20	25	30	45	50	5 (90.0%)
8	30	30	40	40	70	70	0 (100%)
Mean	16.875	21.25	34.375	40	51.25	61.25	10 (83.6%)
*P* value							0.008

Participant	Supination (Operative)	Supination (Nonsurgical)	Pronation (Operative)	Pronation (Nonsurgical)	Supination-Pronation arc (Operative)	Supination-Pronation arc (Nonsurgical)	Supination-Pronation Arc Difference

1	80	90	90	90	170	180	10 (94.4%)
2	75	80	90	90	165	170	5 (97.1%)
3	90	90	90	90	180	180	0 (100%)
4	80	70	90	90	170	160	(-) 10 (106.3%)
5	80	80	90	90	170	170	0 (100%)
6	75	80	80	90	155	170	15 (91.2%)
7	85	80	90	90	175	170	(-) 5 (102.9%)
8	90	90	90	90	180	180	0 (100%)
Mean	81.875	82.5	88.75	90	170.625	172.5	1.875 (99%)
*P* value							0.818

**Figure 2:**
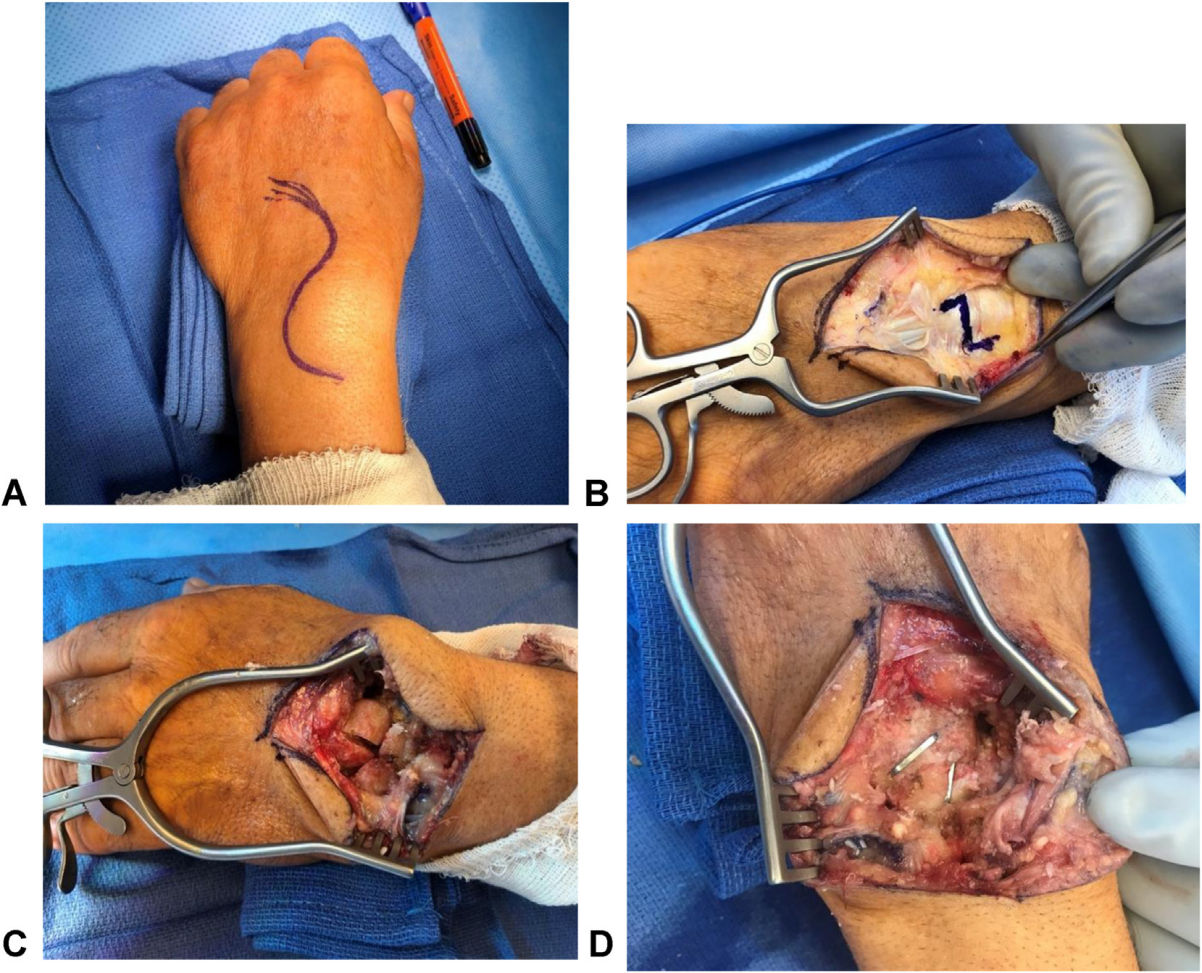
Surgical technique. **A** Marking for a modified dorsal approach to the wrist to maximize exposure. **B** Dorsal approach to wrist with marking for Z plasty of the extensor retinaculum for future repair at the end of the case. **C** After completion of capsulotomy and visualization of the carpals involved in the four-corner fusion. This is after excision of the scaphoid. **D** Final view of the four-corner fusion mass after placement of bone graft and DynaNite Nitinol Staple (Arthrex Inc).

The same surgical technique was performed in all patients included in our study by a single American Board of Orthopaedic Surgery board-certified fellowship trained Orthopedic Hand Surgeon (B.R.). Patients were placed in the supine position with the operative extremity resting on a hand table. A tourniquet was applied to the upper arm and the extremity was prepared with chloraprep solution per hospital protocol. A standard dorsal approach to the wrist was performed (Fig. 2A). The fourth dorsal wrist compartment was entered, and the posterior interosseous nerve was identified and excised in all patients to assist with postoperative pain (Fig. 2B). The four carpals to be fused, which included the capitate, hamate, lunate, and triquetrum, were then denuded of cartilage (Fig. 2C). The wound was irrigated, and allograft (Arthrocell Arthrex Inc) bone was placed in between the prepared carpals. Allograft was used rather than autograft to create a uniform graft for all patients in the study and to minimize any differences in individual patient bone characteristics acting as a confounding variable. At least two staples were used in every case, and whether two or three staples were used was determined intraoperatively by the individual patients anatomical constraints (Fig. 1A and B). A trough was then created along the intended final position of the staple to allow for more seated placement, preventing any impingement from occurring during wrist motion (Fig. 2D). Additionally the legs of the luno-capitate staple was cut 1–2 mm short to minimize any potential of prominence within the carpal tunnel. Finally, a radial styloidectomy was performed for all patients in order to improve range of motion.

## Results

A total of eight patients (n = 53 years of age) agreed to take part in midterm follow-up with a mean follow-up of 27 months (range 9–42 months). Results were measured during postoperative follow-up appointments. When comparing wrist flexion-extension, radial-ulnar deviation, and supination-pronation arcs of motion, patients were found to have maintained 76%, 83%, and 99% of motion, respectively, within these arcs of motion when compared to the contralateral side, respectively. Similar results were seen with key pinch, three-point pinch, and grip strength testing, with maintained strengths of 95%, 89%, and 86%, respectively, all compared to the contralateral side. Regarding patient-reported outcomes in the postoperative period, the mean *Quick*DASH score was 17, the mean overall Michigan Hand Outcomes score in the operative group was 87% compared to the contralateral side, and the visual analog pain scale had a median of 2 (range 0–6). Patients were overall satisfied with their procedure based on the results of the seven-level Likert Satisfaction Scale (median = 6; range 5–7). Radiographic imaging showed that all patients had undergone union of their four-corner arthrodesis with intact Nitinol staples, and there were no complications with hardware noted on radiographs.

## Discussion

A four-corner fusion is another motion sparing alternative used to treat osteoarthritis secondary to SLAC or SNAC wrists, rather than proximal row carpectomy.^13^ Studies have demonstrated that it is a reliable surgical technique able to achieve good long-term patient satisfaction and survivorship.^14^ To achieve this fusion, as previously mentioned, there are a variety of surgical techniques, including but not limited to Kirschner wires (K-wires), locking plates, headless compression screws, and staples.

The use of staples is a relatively newer technique that does demonstrate potential for improved outcomes. A case report by Gaston^15^ was published in 2020, where he performed a scaphoid excision with isolated capitolunate fusion using Nitinol staples for a 38-year-old man with stage II SLAC wrist arthritis. The patient went on to successful union as evidenced by computed tomography scan at 6 weeks, with complete relief of pain and no change in his preoperative range of motion. Another study demonstrated the use of a quadtripodal shape memory staple with mean follow-up of 7.4 years in 41 patients demonstrated notable pain relief and range of motion over 90% fusion rates.^16^ Another report of compression staple has been shown to achieve an isolated capitolunate fusion in a patient with a SNAC wrist.^17^ Finally, some of the initial complications with use of staples, dorsal impingement or prominent legs, can be mitigated through simple surgical techniques that do not undermine the biomechanical properties of the construct.^7^ However, these studies lack a larger patient size as well as a detailed surgical technique with modifications.

As previously mentioned, the use of DynaNite Nitinol Staple (Arthrex Inc) in the wrist for four-corner arthrodesis is a relatively newer technique with scarce data in the literature to support its use. Although we did not have long-term follow-up and, more notably, only had eight patients all of whom were male patients to extrapolate data from, we believe this is a step in the right direction to advance the field of orthopaedic hand surgery with this specific surgical procedure. Additionally, by implementing intraoperative techniques such as simultaneously performing radial styloidectomy, minimizing dorsal staple prominence, and shortening the legs of the staples, we were able to avoid the proposed complications of dorsal impingement and hardware irrigation without compromising fusion. Further studies with larger sample sizes and long-term follow-up are necessary and would provide further evidence to support the use of DynaNite Nitinol Staple (Arthrex Inc) in the wrist for four-corner fusions.

There are several limitations to our study. Given the retrospective nature of this data collection, preoperative parameters included above, such as grip strength and range of motion, were not able to be collected. However, this was mitigated by using the patients contralateral noninvolved hand as a control. Furthermore, this study was also limited in terms of sample size, as only eight total patients were able to participate. There were also patients that were loss to follow-up, where fourteen patients had met inclusion criteria but only eight were able to participate. Despite lacking preoperative values or an alternative construct as a control, patients were overall satisfied with their surgical outcome and functioning similarly when compared with the contralateral extremity, particularly in terms of pronation-supination, key pinch, three-point pinch, and grip strength.

In conclusion, patients undergoing four-corner wrist fusion using DynaNite Nitinol Staple (Arthrex Inc) with the modifications described above were satisfied with their procedure and had minimal wrist pain at baseline and with activities of daily living. Patients demonstrated that they had functional range of motion of the wrist with strength that was comparable to the nonsurgical extremity. The results of this study show that midterm outcomes of four-corner fusions performed with DynaNite Nitinol Staple (Arthrex Inc) along with intraoperative modifications have promising results in overall functional and patient-reported outcomes. In addition, this construct offers reliable fixation for achieving union of the four-corner arthrodesis. Considerable differences were found between flexion-extension, radial-ulnar deviation, and the overall Michigan Hand Outcomes score, indicating that patients had more motion on the contralateral extremity and subjectively improved patient-reported outcomes in terms of hand function, activities of daily living, and pain.

## Conflicts of Interest

Dr Riedel is a consultant for Arthrex but has no has no financial or conflicts of interest to disclose pertinent to this study. No benefits in any form have been received or will be received by the other authors related directly to this article.
